# Dose–Response Effect of Consuming Commercially Available Eggs on Wintertime Serum 25-Hydroxyvitamin D Concentrations in Young Australian Adults: a 12-Week Randomized Controlled Trial

**DOI:** 10.1093/jn/nxac044

**Published:** 2022-02-26

**Authors:** Robin M Daly, Belinda De Ross, Jenny Gianoudis, Sze-Yen Tan

**Affiliations:** Institute for Physical Activity and Nutrition (IPAN), School of Exercise and Nutrition Sciences, Deakin University, Burwood, Melbourne, Victoria, Australia; Institute for Physical Activity and Nutrition (IPAN), School of Exercise and Nutrition Sciences, Deakin University, Burwood, Melbourne, Victoria, Australia; Institute for Physical Activity and Nutrition (IPAN), School of Exercise and Nutrition Sciences, Deakin University, Burwood, Melbourne, Victoria, Australia; Institute for Physical Activity and Nutrition (IPAN), School of Exercise and Nutrition Sciences, Deakin University, Burwood, Melbourne, Victoria, Australia

**Keywords:** eggs, vitamin D, serum 25(OH)D, winter, young adults, serum lipids

## Abstract

**Background:**

Vitamin D deficiency is a common health concern during winter. Eggs are one of the few rich dietary sources of vitamin D, containing cholecalciferol (vitamin D-3) and 25-hydroxyvitamin D-3 [25(OH)D_3_], with the latter reported to be 5 times more potent at increasing serum 25(OH)D concentrations, the major circulating form of vitamin D. However, whether there is an optimal dose of eggs to increase or maintain 25(OH)D concentrations during wintertime is not known.

**Objectives:**

To evaluate the dose–response effect of consuming 2, 7, or 12 commercially available eggs per week on serum 25(OH)D concentrations during the autumn-winter months in young adults. Secondary aims were to investigate changes in serum lipids, and the feasibility (adherence) and acceptability to consuming the eggs.

**Methods:**

In a 12-wk randomized controlled trial, 51 adults aged 25–40 y were randomly assigned to consume 2 eggs/wk (control, *n* = 17), 7 eggs/wk (*n* = 17), or 12 eggs/wk (*n* = 17). Change in serum 25(OH)D was the primary outcome as assessed by LC/MS/MS. Serum lipids were assessed using standard techniques, and acceptability to consuming the eggs was assessed via a questionnaire.

**Results:**

Forty-two (82%) participants completed the study. Mean adherence to the eggs was 83% for controls, 86% for 7 eggs/wk, and 83% for 12 eggs/wk. Mean serum 25(OH)D concentrations did not change significantly in either the 7-eggs/wk (−8.3 nmol/L; 95% CI: −17.0, 0.4 nmol/L) or 12-eggs/wk (−7.2 nmol/L; 95% CI: −18.6, 4.3 nmol/L) groups, but decreased by 28.6 nmol/L (95% CI: −38.1, −18.9 nmol/L) in controls, which led to a significant (*P* = 0.003) between-group difference for the change after 12 wk. Serum lipids did not differ between the groups, and acceptability profiles to consuming the eggs were positive and similar for all 3 groups.

**Conclusions:**

Consuming 7 commercially available eggs per week for 12 wk was effective for attenuating the wintertime decline in circulating vitamin D concentrations in young Australian adults, with 12 eggs/wk not providing any additional benefits.

## Introduction

Vitamin D deficiency is recognized as a prevalent public health problem globally (1, 2). In Australia, the 2011–12 National Health Survey revealed that 20% of adults (∼3.29 million people based on consensus data) aged ≥25 y were vitamin D deficient [25-hydroxyvitamin D (25(OH)D) <50 nmol/L], with the highest rates of deficiency in adults aged 25–34 y (3). This is likely due in part to the greater use of vitamin D–containing supplements in older Australian adults (4). Given the importance of vitamin D to bone health and possibly other nonskeletal conditions, there is a need to promote strategies to maintain adequate vitamin D concentrations throughout life, particularly in young adults and during winter when serum 25(OH)D concentrations decrease by ∼15–20 nmol/L and the risk of vitamin D deficiency more than doubles (3, 5).

The main source of vitamin D is through sun exposure, but due to geographical differences, seasonal changes in UV radiation, differences in skin pigmentation, lifestyle habits, and adherence to sun protection messages, it can be difficult to achieve adequate year-round exposure to maintain sufficient serum 25(OH)D concentrations. In addition, because few foods naturally contain vitamin D many adults have habitual intakes below the dietary reference or adequate intake (AI) values, which typically range from 5 to 15 μg/d (200 to 600 IU/d) (6–8) or ≤20 μg/d (800 IU/d) for adults aged >70 y (8). Of the limited food sources that contain vitamin D (e.g., oily fish, meat, eggs, dairy), eggs have significant quantities of both cholecalciferol (vitamin D-3) and the hydroxylated form of vitamin D-3, 25(OH)D_3_, which can be up to 5 times more potent in raising serum 25(OH)D concentrations than cholecalciferol (9, 10). As a result, there has been interest in whether eggs can play a key role in maintaining circulating 25(OH)D concentrations (11), but the optimal dose required to increase or maintain 25(OH)D concentrations is not known.

Several studies measuring the vitamin D concentrations of Australian eggs have reported the cholecalciferol and 25(OH)D_3_ content (free-range and cage eggs) per egg (60 g) to be 0.4–0.8 μg and 0.4–0.6 μg, respectively (12, 13). Assuming 25(OH)D_3_ is 5 times more bioactive than cholecalciferol, which is a correction factor included in some vitamin D food composition databases to provide a more accurate estimate of vitamin D intakes, the total vitamin D activity of eggs would be 2.5–3.4 μg per egg (∼100–135 IU). Based on a standard 120-g serve of eggs (2 large eggs), this dose would be equivalent to the current AI of 5 μg/d for vitamin D for Australians aged 1–50 y (7). Previous research has shown that serum 25(OH)D concentrations increase by ∼1–2 nmol/L for every additional 100 IU of vitamin D-3 (14–16). Because the current Australian Dietary Guidelines recommend eating ≤7 eggs/wk as part of a healthy, balanced diet (17), it is feasible that this weekly dose would be sufficient to maintain or attenuate the wintertime decrease in serum 25(OH)D concentrations. Indeed, the findings from an 8-wk randomized controlled trial (RCT) in 55 adults aged 45–70 y residing in Ireland revealed that weekly consumption of 7 vitamin D-3–enriched eggs or seven 25(OH)D-enriched eggs was equally effective at maintaining serum 25(OH)D concentrations compared with consumption of ≤2 eggs/wk, with the latter experiencing a significant mean 6.4-nmol/L wintertime reduction in circulating 25(OH)D concentrations (11). In this study, habitual vitamin D intakes were ∼6.0–6.9 μg/d, and consumption of the vitamin D-3–enriched eggs or 25(OH)D-enriched eggs provided an additional 3.5 and 4.5 μg vitamin D (total vitamin D activity), respectively, which yielded a total intake of ∼10–11 μg/d (11). Although these findings provide some evidence to support the weekly consumption of 7 vitamin D–fortified eggs for maintaining wintertime vitamin D status, no studies have investigated whether there is a dose-–response relation between egg consumption and serum 25(OH)D concentrations. This is important to determine whether there might be a minimum dose of eggs that is required to maintain or increase circulating 25(OHD) concentrations.

The primary aim of this 12-wk RCT was to compare the effects of consuming 2, 7, and 12 commercially available eggs per week on serum 25(OH)D concentrations during the autumn-winter months in adults aged 25–40 y residing in southern Australia. Secondary aims were to investigate the effects of the intervention on serum lipids, and the feasibility (adherence) and acceptability to consuming the different doses of eggs.

## Methods

### Study design

This was a 12-wk, 3-arm RCT in which 51 men and women aged 25–40 y were randomly allocated (1:1:1 ratio) to consume: *1*) 2 eggs/wk (control group, *n* = 17); *2*) 7 eggs/wk (*n* = 17), or *3*) 12 eggs/wk (*n* = 17). Randomization was at the level of the individual participant in blocks of 3 using a computer-generated random number sequence by an independent researcher. The intervention was conducted from May (late autumn) to August (winter) 2021 in Melbourne, Australia. All baseline and follow-up assessments were performed at a local pathology clinic (blood collection) or online (questionnaires), and participants and the research staff conducting the trial were not blinded to the group allocation. However, those handling the blood samples and conducting the blood analyses were blinded to the group allocation. The trial was managed through the Institute for Physical Activity and Nutrition at Deakin University, Burwood, Melbourne, Australia. The study was approved by the Deakin University Human Research Ethics Committee (HREC 2020-159) and registered at the Australian and New Zealand Clinical Trials Registry (www.anzctr.org.au/) as ACTRN12620001057976. Written informed consent was obtained from all participants prior to commencing the study.

### Participants

**FIGURE 1:**
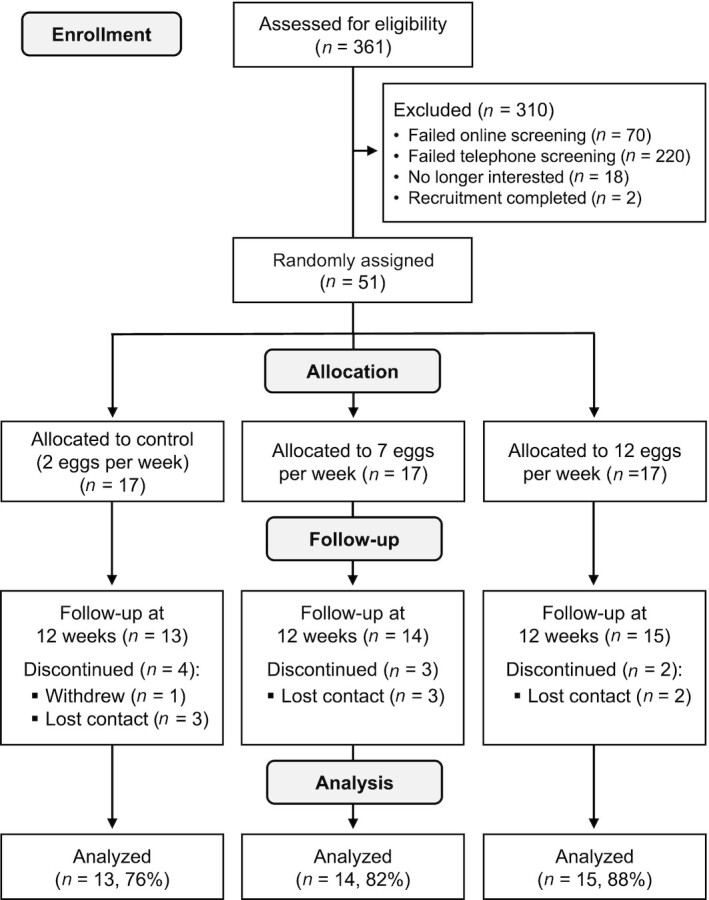
Flowchart of participants through the study.

Healthy men and women aged 25 to 40 y residing in the community were recruited from metropolitan Melbourne and Geelong (latitude ∼38°S) in Victoria, Australia. Interested participants were first screened via an online survey in which they progressed to stage 2 (telephone screening) if they were aged 25–40 y, not allergic to eggs, a nonsmoker, had an occupation or lifestyle over the past 3 mo that involved spending on average <3 h/d outdoors between 10:00 and 14:00, and were currently not taking (or in the past 3 mo) vitamin D supplements or multivitamin supplements containing a vitamin D dose >200 IU. Eligible participants were then contacted by the research staff and deemed ineligible based on the following: regular consumers of whole eggs (>3/wk on average over the past 3 mo); currently participating in a weight-loss or dietary-based program; currently taking corticosteroids (oral), glucocorticoids, anticonvulsants, or thiazide diuretics; current diagnosis of cancer (or in past 6 mo), diabetes, kidney disease, or a gastrointestinal disorder that can affect nutrient absorption; cardiovascular event that required hospitalization in the past 3 mo or currently undergoing cardiovascular rehabilitation; alcohol intake >2 standard drinks on ≥5 d/wk; recent (past 3 mo) and proposed holiday or trip (>1 wk) involving a high level (more than usual) of sun exposure; use of tanning facilities in the past 3 mo; unable to commit to the study requirements; or not willing to be randomly allocated to 1 of the 3 groups. A total of 361 adults expressed an interest in participating in the study, of whom 51 were deemed eligible to participate and randomly allocated to 1 of the 3 groups (**Figure 1**).

### Intervention

Participants were randomly assigned to 1 of 3 groups: *1*) a control group in which they were asked to consume 2 eggs/wk; *2*) consumption of 7 eggs/wk in line with the current Australian dietary guidelines; or *3*) consumption of 12 eggs/wk. Due to COVID restrictions on travel all participants were provided with a prepaid gift card and asked to purchase a 3-wk supply of the same brand and size of commercially available eggs from the same major supermarket chain (Woolworths Extra Large Free Range Eggs, ∼60 g/egg) several days prior to starting the study and during weeks 3, 6, and 9. All participants were required to send an electronic version (photo) of their receipt to confirm purchase of the correct type and dose of eggs. They were encouraged to eat the eggs in whole form where possible (e.g., boiled, scrambled, poached, fried) and not as part of shared meals, not to consume any nonstudy eggs, and to maintain their typical diet (eating) habits throughout the study. Adherence to the eggs was determined from a daily eggs compliance calendar that was placed on the participant's fridge via a magnet strip. Overall egg compliance was calculated as the percentage of the eggs consumed relative to the total prescribed.

### Assessment of the vitamin D content of the eggs

The cholecalciferol and 25(OH)D_3_ content of the eggs was assessed at baseline and week 6 at the National Measurement Institute of Australia, Port Melbourne, Victoria, which is accredited by the National Association of Testing Authorities (NATA) for analyzing vitamin D in foods. At each timepoint, 3 batches of free-range eggs (60 g/egg with 3 eggs from each batch combined for analysis) purchased from 3 different supermarkets (from the same major chain—maximum 10,000 birds/ha stocking density) in the north, east, and west of Melbourne were assessed to reflect the broad location (place of residence) of the participants enrolled in the study. The cholecalciferol and 25(OH)D_3_ content of the eggs (raw) was assessed by HPLC with photodiode array (18). The vitamin D content (expressed as micrograms per 60-g egg) of each batch of eggs was averaged to give a mean (± SD) value of cholecalciferol and 25(OH)D_3_ at baseline and week 6. Because previous research has shown that each microgram of orally consumed 25(OH)D_3_ is 5 times more effective in raising serum 25(OH)D concentrations than an equivalent amount of cholecalciferol (9), the total vitamin D content of the eggs (expressed as both micrograms per egg and international units per egg) was calculated as follows: cholecalciferol + [5 × 25(OH)D_3_].

### Serum 25(OH)D and serum lipids

Fasted, resting morning venous blood samples were collected at baseline and postintervention from each participant's antecubital vein at local pathology clinics around Melbourne, and sent to a central NATA-accredited pathology laboratory for processing. Total serum cholesterol, HDL cholesterol, and triglycerides were assessed using an enzymatic, colorimetric method (Roche Cobas c701 analyzer). LDL cholesterol was calculated by using the Friedewald formula. Serum aliquots were stored at −70°C for the assessment of serum 25(OH)D by a validated LC/MS/MS method, using a Shimadzu Nexeria ultra performance LC system for separation, and a Sciex Triple Quad 5500 system for detection, at Monash Health Pathology. All samples were assessed in the same batch (in duplicate) at the completion of the study. The calibrators for the LC/MS/MS 25(OH)D method are traceable to the National Institute of Standards and Technology Standard Reference material 972a. The intra-assay CV was 5.7% at 32.3 nmol/L, 3.9% at 60.6 nmol/L, and 2.7% at 101.8 nmol/L, and the interassay CV was 7.8% at 41.1 nmol/L and 7.0% at 113 nmol/L.

### Demographics, anthropometrics, medical history, skin type, and adverse events

Height and weight were self-reported because participants were unable to attend Deakin University due to COVID restrictions. Information on demographics (age, gender, country of birth, educational background), use of lipid-lowering or other medications known to influence vitamin D metabolism (e.g., antibiotics, anticonvulsants), and dietary supplements was obtained from a health and lifestyle questionnaire. Country of birth was used to classify participants as either Europid (born in Australia, New Zealand, Northern Europe, Canada, United States) or non-Europid (Italy, Vietnam, China, India, Philippines, Malaysia, Hong Kong, Pakistan, Indonesia, or Singapore). Education was classified as either completed high school/technical/trade certificate or having a university or tertiary level qualification. The Fitzpatrick skin type scale (19) was used to categorize participants’ skin type by their response to sun exposure: Type I: pale-white skin, always burns, never tans; Type II: white or fair skin, usually burns, tans minimally; Type III: light-brown skin, sometimes mild burn, tans uniformly; Type IV: moderate-brown skin, rarely burns, always tans well; Type V: dark-brown, moderately pigmented brown skin, very rarely burns, tans very easily; Type VI: black deeply pigmented dark-brown to black skin; never burns, tans very easily. Participants were asked to report any alterations to, or new, medications or use of supplements at follow-up. Any adverse events associated with eating the eggs were determined via an online questionnaire that the participants completed every 3 wk.

### Diet

Nutrient intakes were assessed at baseline and follow-up from two 24-h food records (1 weekday and 1 weekend day) using the Automated Self-Administered 24-Hour Dietary Assessment Tool (ASA24, version Australia 2016), developed by the National Cancer Institute, Bethesda, MD, United States (20). The ASA24 links food items to the AUSNUT 2011–13 database and automatically records all food and beverage details, including a description of the foods, brand names, and serving size estimates (in cups, teaspoons, pieces, slices, etc., as standard for the food or beverage). The assessment of dietary vitamin D intake was not possible in this study because vitamin D values are not included in AUSNUT 2011–13 database. Dietary data were missing (record not completed) or incomplete for 2 participants at baseline (*n* = 1, control; *n* = 1, 7 eggs/wk) and 5 participants at the 12-wk follow-up (*n* = 2, control; *n* = 1, 7 eggs/wk; *n* = 2, 12 eggs/wk).

### Physical activity

Self-reported time spent in moderate-vigorous physical activity (MVPA) was assessed at baseline and follow-up using the Active Australia survey (21, 22). Participants were asked about the number of days and duration (time) they walked for recreation or transport and did any moderate or vigorous physical activities in the last 7 d. Total MVPA (minutes per week) was computed as the sum of walking, moderate, and vigorous physical activity. One participant in the 7-eggs/wk group did not complete the survey at the 12-wk follow-up.

### Sun exposure and protection practices

To provide an estimate of sun exposure habits throughout the study, participants were asked at baseline and every 3 wk throughout the study (via an online questionnaire developed for this study) about the time (<15, 15–30, 30–60, or >60 min/d) they usually spent outdoors between 10:00 and 14:00 (the peak UV radiation period during May to August in Melbourne) on both weekdays and weekend days in the past 3 wk. Participants were also asked about their sun protection practices when outdoors, including use/wearing of a broad-brimmed hat, cap, or other head covering, wearing of a long-sleeve shirt and long pants, and use of sunscreen. Those who responded yes to these sun protection practices more than half the time or always/almost always were classified as users of sun protection practices.

### Egg acceptability

Egg consumption acceptability was evaluated at the end of the study using a modified version of the Food Acceptability Questionnaire (23). For this study, the questionnaire consisted of 7 questions scored on a 7-point response scale in which participants were asked to rate whether they liked eating the eggs, whether they liked the taste of eggs, how satisfied they felt after eating the eggs, how easy/difficult it was for them to prepare the eggs to eat, the level of effort required to eat the eggs, whether eating out influenced regular egg consumption, and the ease with which they could continue to eat the same number of prescribed eggs after completion of the study. Data were missing for 4 participants (*n* = 1, control; *n* = 2, 7 eggs/wk; *n* = 1, 12 eggs/wk) who did not complete/return the eggs consumption acceptability questionnaire.

### Sample size

The sample size calculations are based on the expected difference for the change in serum 25(OH)D concentrations between the 7- and 12-eggs/wk groups relative to controls (2 eggs/wk). The sample size was based on the findings from Hayes et al. (11) who reported that consumption of 7 eggs/wk (containing 3.5–4.5 μg/egg) maintained serum 25(OH)D concentrations during wintertime compared with controls (≤2 eggs/wk), who experienced a mean 6.4 ± 6.7 nmol/L decrease after 8 wk. Our previous findings from a 16-wk study in middle-aged women followed during the winter months in Melbourne (Australia) also found that serum 25(OH)D concentrations decreased by an average of 12 nmol/L after 4 mo (24). Based on the above findings, we estimated that a sample size of 51 participants (17 per group) would provide 90% power (2-tailed, *P* < 0.05) to detect an 8- and 14-nmol/L net difference between the control (2 eggs/wk) and 7- and 12-eggs/wk groups, respectively, using a more conservative SD of 10. These sample size calculations take into account a potential 20% attrition after 12 wk.

### Statistical analysis

All statistical analysis was conducted using SPSS for Windows (version 26; SPSS Inc). All data were analyzed using an intention-to-treat approach, with every randomized participant included in the analyses. Sensitivity analysis (per protocol) was also performed by only including participants with ≥80% adherence to the eggs. Normality of data was tested via visual inspection of frequency distributions and the use of the Kolmogorov–Smirnov tests. All serum lipid measures were log transformed due to nonnormality. Descriptive statistics are reported as mean ± SD or number with proportion (percentage), and all change data are reported as means with 95% CIs, unless stated. ANCOVA was used to test for between-group differences for the absolute changes over 12 wk, adjusting for the baseline variable being tested, age, and sex, with Bonferroni-adjusted *t* tests used for post hoc analysis. For the primary outcome of serum 25(OH)D, additional analysis was performed that also included country of birth, physical activity, and sun exposure as covariates. Sensitivity analysis was also undertaken excluding any participants who commenced taking vitamin D supplements during the study for >1 wk. Paired *t* tests were used to test for within-group changes over time. For any missing data, no imputation was performed. χ^2^ tests were used to test for group differences in the responses to the egg acceptability questions, with results reported as median and IQR. Statistical significance was set at *P* < 0.05.

## Results

### Vitamin D content of the eggs

The mean ± SD vitamin D content of the eggs (per 60-g egg) used throughout the study was 0.71 ± 0.25 μg/egg for 25(OH)D_3_, 1.63 ± 0.70 μg/egg for cholecalciferol, and 5.18 ± 1.60 μg/egg for total vitamin D (**Supplemental Table 1**). Based on the total vitamin D concentrations of the eggs, the prescribed weekly vitamin D doses for the control (2 eggs/wk), 7-, and 12-eggs/wk groups were 10.4 μg (414 IU), 36.3 μg (1450 IU), and 62.2 μg (2486 IU), respectively.

### Baseline characteristics

As shown in **Table 1**, the average age of the participants was ∼33 y, 75% were female, 41% were overweight or obese, 71% were classified as Europid, and the vast majority (86%) had a serum 25(OH)D concentration ≥50 nmol/L at baseline (mean 79 nmol/L). No participants were taking lipid-lowering medication or other medication(s) known to influence vitamin D metabolism.

**TABLE 1:** Baseline characteristics of the young adults allocated to the 2- (control), 7-, and 12-eggs/wk groups^1^

Characteristic	Control	7 eggs/wk	12 eggs/wk
*n*	17	17	17
Men | women, n	3 | 14	5 | 12	5 | 12
Age, y	33.4 ± 5.4	32.7 ± 5.0	32.7 ± 5.5
Height, cm	165.0 ± 10.1	168.5 ± 10.8	169.7 ± 9.8
Weight, kg	67.0 ± 12.5	76.1 ± 25.4	71.5 ± 15.4
BMI, kg/m^2^	24.5 ± 3.9	26.6 ± 8.2	24.8 ± 4.6
Healthy (BMI 18 to <25), *n* (%)	10 (59)	10 (59)	10 (59)
Overweight (BMI 25–29.9), *n* (%)	6 (35)	5 (29)	5 (29)
Obese (BMI ≥30), *n* (%)	1 (6)	2 (12)	2 (12)
Country of birth,^2^*n* (%)			
Europid	12 (71)	11 (65)	13 (77)
Non-Europid	5 (29)	6 (35)	4 (23)
Skin type,^3^*n* (%)			
Type I–II	11 (65)	6 (35)	10 (59)
Type III–IV	6 (35)	10 (59)	7 (41)
Type V–VI	0 (0)	1 (6)	0 (0)
Highest level of education, *n* (%)			
High school or trade certificate	4 (23)	1 (6)	3 (18)
University or higher	13 (77)	16 (94)	14 (82)
Dyslipidemia,^4^*n* (%)	3 (18)	2 (12)	4 (24)
Vitamin D status			
Insufficient (<75 nmol/L), *n* (%)	10 (59)	8 (47)	9 (53)
Deficient (<50 nmol/L), *n* (%)	1 (6)	4 (24)	2 (12)

1 Values represent number and percentage or mean ± SD.

2 Europid includes those born in Australia, Canada, New Zealand, Northern Europe, and the United States; non-Europid includes those born in southern Europe, Asia, the Middle East, India and Sri Lanka, Pacific Islands, Africa, South and Central America, Aboriginal Australians, and Torres Strait Islanders.

3 Fitzpatrick skin type scale: Type I: pale-white skin, always burns, never tans; Type II: white or fair skin, usually burns, tans minimally; Type III: light-brown skin, sometimes mild burn, tans uniformly; Type IV: moderate-brown skin, rarely burns, always tans well; Type V: dark-brown, moderately pigmented brown skin, very rarely burns, tans very easily; Type VI: black deeply pigmented dark-brown to black skin; never burns, tans very easily.

4 Dyslipidemia: cholesterol ≥5.5 mmol/L, or HDL cholesterol <1.0 mmol/L, or LDL cholesterol ≥3.5 mmol/L, or triglycerides ≥2.0 mmol/L.

### Study attrition, egg adherence, and adverse events

Overall, 9 (18%) of the participants did not complete the 12-wk follow-up assessment (controls, *n* = 4; 7 eggs/wk, *n* = 3; 12 eggs/wk, *n* = 2) (Figure 1). The reasons for withdrawal or lack of follow-up included: lost contact (*n* = 8) and withdrew with no reason stated (*n* = 1). Participants with no follow-up assessment did not differ significantly in age, height, weight, physical activity, serum 25(OH)D, or serum lipids from those who completed the intervention (data not shown). The mean ± SD (range) adherence to the eggs for all participants (*n* = 51) was 84 ± 32% (0, 100) and did not differ significantly (*P* = 0.94) between the control [83 ± 33% (0, 100)], 7-eggs/wk [86 ± 30% (0, 100)], and 12-eggs/wk [83 ± 33% (0, 100)] groups. Five participants (controls, *n* = 2; 7 eggs/wk, *n* = 1; 12 eggs/wk, *n* = 2) did not consume any eggs and all did not complete the 12-wk follow-up assessment. For the 42 participants who completed the study, mean adherence to the eggs was 96 ± 9% (range 54, 100) and similar between the groups: controls = 98%; 7 eggs/wk = 98%; 12 eggs/wk = 94%. One minor adverse event (gastrointestinal discomfort) was reported by 1 participant in the 12-eggs/wk group.

### Weight, physical activity, diet, and supplement use

There were no within-group changes nor between-group differences for weight, MVPA, or diet throughout the study, with the exception that total protein intake increased on average by 14 g (*P* < 0.029) in the 12-eggs/wk group (**Supplemental Table 2**). At baseline and follow-up, 37% and 38% of all participants reported consuming alcohol (mean intake 19 g and 18 g at baseline and follow-up, respectively), with no differences between the groups (baseline, *P* = 0.485; week 12, *P* = 0.729). One participant from both the 7- and 12-eggs/wk groups reported taking vitamin D supplements during the study [7 eggs/wk, 200 IU/d from week 2; 12 eggs/wk, 1000 IU/d from week 4).

### Serum 25(OH)D concentrations

**FIGURE 2:**
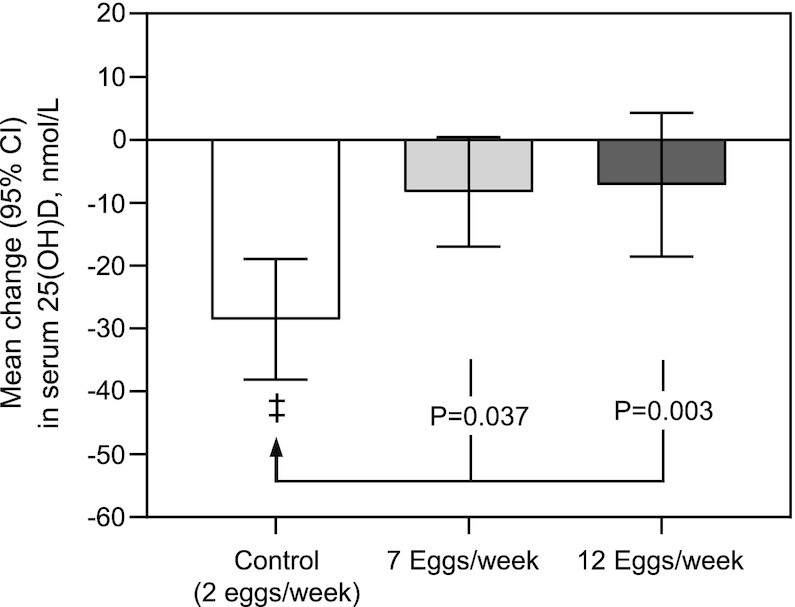
Unadjusted absolute changes in serum 25(OH)D concentrations in young adults after 12 wk of consuming 2 (control), 7, or 12 eggs/wk. Values are mean (95% CI). Controls: *n* = 13; 7 eggs/wk: *n* = 14; 12 eggs/wk: *n* = 15. Between-group differences were assessed using ANCOVA adjusted for baseline values, age, and sex, with the *P* values based on Bonferroni-adjusted post hoc tests. Paired *t* tests were used to assess within-group changes over time, ^‡^Significantly different compared with baseline; *P* < 0.001. 25(OH)D, 25-hydroxyvitamin D.

Mean serum 25(OH)D concentrations decreased nonsignificantly by 8.3 nmol/L (*P* = 0.06) and 7.2 nmol/L (*P* = 0.20) in the 7- and 12-eggs/wk groups, respectively, after 12 wk. In contrast, controls had a significant 29-nmol/L mean reduction (*P* < 0.001) in 25(OH)D concentrations, which led to a significant (*P* = 0.003) between-group difference for the change after 12 wk (**Table 2**, **Figure 2**). Post hoc analysis revealed that changes in the controls were significantly different from both the 7- and 12-eggs/wk groups, in which the changes were similar (Table 2, Figure 2). All results remained unchanged after also including country of birth, physical activity, and sun exposure as covariates (*P* = 0.005). Similar results were also found after excluding the 2 participants who commenced taking vitamin D supplements during the trial (*P* = 0.002), with the exception that mean serum 25(OH)D concentrations decreased significantly from baseline to 12 wk in both the 7-eggs/wk (mean change = 9.7 nmol/L; *P* = 0.036) and 12-eggs/wk (10.7 nmol/L; *P* = 0.027) groups.

**TABLE 2:** Mean baseline values and changes after 12 wk in young adults for weight, serum 25(OH)D, and serum lipid concentrations in the 2- (control), 7-, and 12-eggs/wk groups^1^

	Control		7 eggs/wk		12 eggs/wk		
	Baseline (*n* = 17)	Change^2^ (*n* = 13)	Baseline (*n* = 17)	Change^2^ (*n* = 14)	Baseline (*n* = 17)	Change^2^ (*n* = 15)	*P* value^3^
Weight, kg	67.0 ± 12.5	0.3 (−1.0, 1.6)	76.1 ± 25.4	0.0 (−0.9, 0.9)	71.5 ± 15.4	0.7 (−0.1, 1.4)	0.561
Serum 25(OH)D, nmol/L	84.4 ± 28.7	−28.6 (−38.1, −18.9)**	74.2 ± 28.1	−8.3 (−17.0, 0.4)^a^	79.5 ± 25.1	−7.2 (−18.6, 4.3)^b^	0.003
Total cholesterol, mmol/L	4.79 ± 1.21	0.22 (−0.07, 0.52)	4.63 ± 0.86	0.27 (−0.15, 0.70)	4.68 ± 0.99	0.47 (0.09, 0.84)*	0.440
HDL cholesterol, mmol/l	1.56 ± 0.42	−0.01 (−0.08, 0.07)	1.35 ± 0.20	0.07 (−0.07, 0.20)	1.62 ± 0.33	0.05 (−0.07, 0.16)	0.721
LDL cholesterol, mmol/L	2.81 ± 1.19	0.21 (−0.04, 0.46)	2.73 ± 0.65	0.16 (−0.19, 0.52)	2.64 ± 0.98	0.41 (0.05, 0.76)*	0.444
Triglycerides, mmol/L	0.95 ± 0.42	0.02 (−0.12, 0.17)	1.21 ± 0.78	0.07 (−0.13, 0.29)	0.91 ± 0.37	0.03 (−0.14, 0.21)	0.888

1 All baseline values are unadjusted means ± SD. 25(OH)D, 25-hydroxyvitamin D.

2 All within-group changes from baseline are unadjusted means (95% CI) and were assessed by paired *t* tests. ^*,**^Significantly different from baseline: *P < 0.05; **P < 0.001.

3 *P* values for between-group differences were assessed using ANCOVA adjusted for baseline values, age, and sex, with Bonferroni-adjusted *t* tests used for post hoc analysis. ^a,b^Significantly different from change in controls: ^a^*P* < 0.05, ^b^*P* < 0.01.

### Serum lipids

There were no between-group differences or within-group changes for any serum lipid measure, with the exception that total cholesterol (*P* = 0.017) and LDL cholesterol (*P* = 0.036) increased significantly after 12 wk in the 12 eggs/wk group (Table 2).

### Sun exposure and protection practices

On average, the vast majority (71–82%) of participants in all 3 groups reported that they spent on average ≤30 min outdoors on the weekday between 10:00 and 14:00 throughout the entire 12-wk study period. Although the time spent outdoors was greater on the weekends, 69–82% of all participants reported that they spent ≤60 min outdoors on the weekend (**Supplemental Table 3**). There were no group differences in sun exposure habits at any time throughout the study, with the exception that participants in the control and 7-eggs/wk groups tended to spend greater time outdoors on the weekend at week 6 and during the weekdays at week 12 (Supplemental Table 3). Overall, 86–100% of participants reported that they undertook safe sun protection practices all at times throughout the study.

### Eggs acceptability

Overall, participants rated highly that they liked eating the eggs and their taste, and that it was not too difficult or too much effort to consume their prescribed dose of eggs (**Table 3**). There were no significant differences in responses between the 3 groups to any of the egg acceptability questions, but there was a trend for the median scores to the questions *“How well did you like the taste of eggs?”* and *“How easy or difficult was it for you to prepare the eggs to eat?”* to be lower in the 12-eggs/wk group (both *P* = 0.06).

**TABLE 3:** Egg consumption acceptability as rated on a food acceptability questionnaire in young adults at the end of the 12-wk intervention in all participants and those in the 2- (control), 7-, and 12-eggs/wk groups^1^

Acceptability questions	All (*n* = 38)	Control (*n* = 12)	7 eggs/wk (*n* = 12)	12 eggs/wk (*n* = 14)	*P* value^2^
How well did you like eating the eggs? (1 = Not at all, 7 = Extremely)	6.0 (5.0, 7.0)	6.5 (6.0, 7.0)	6.0 (5.0, 7.0)	5.0 (3.8, 5.3)	0.225
How well did you like the taste of the eggs? (1 = Not at all, 7 = Extremely)	6.0 (5.0, 7.0)	7.0 (6.0, 7.0)	6.0 (5.0, 7.0)	5.0 (4.0, 5.3)	0.06
How easy or difficult was it for you to prepare the eggs to eat? (1 = Extremely difficult, 7 = Extremely easy)	7.0 (5.0, 7.0)	7.0 (7.0, 7.0)	7.0 (6.0, 7.0)	4.5 (3.8, 7.0)	0.06
How much effort did it take for you to consume the eggs? (1 = A lot of effort, 7 = No effort at all)	5.0 (3.0, 7.0)	7.0 (3.3, 7.0)	5.0 (3.5, 6.0)	5.0 (2.0, 6.0)	0.135
How satisfied did you feel after eating eggs? (1 = Extremely dissatisfied, 7, Extremely satisfied)	5.0 (5.0, 6.0)	5.5 (5.0, 7.0)	6.0 (5.0, 6.8)	5.0 (4.0, 6.0)	0.492
Did eating out influence your ability to consume the eggs as part this study? (1 = Never, 7, Always)	2.0 (1.0, 5.0)	2.0 (1.0, 5.0)	2.0 (1.0, 4.0)	2.0 (1.0, 5.0)	0.657
How easy could you continue consuming the same number of eggs after completion of the study? (1 = Extremely difficult, 7 = Extremely easy)	4.0 (3.0, 6.3)	5.0 (3.0, 7.0)	5.0 (4.0, 6.8)	4.0 (2.0, 4.3)	0.155

1 All values are median (IQR).

2 *P* values for between-group difference were assessed using χ^2^ tests. One participant from each group did not complete the acceptability questionnaire at follow-up.

### Per protocol analysis

All results (between-group effects) remained unchanged following the per protocol analysis, which included the 38 participants (controls, *n* = 12; 7 eggs/wk, *n* = 13; 12 eggs/wk, *n* = 13) who achieved ≥80% adherence to eggs (data not shown).

## Discussion

The main finding from this 12-wk RCT was that consumption of 7 or 12 commercially available eggs per week was equally effective for attenuating the wintertime decrease in serum 25(OH)D concentrations in young Australian adults. Furthermore, participant acceptability profiles in relation to consuming the eggs were positive, with no significant differences between those consuming 2, 7, or 12 eggs/wk. Finally, there was no effect (group differences) on our secondary outcomes of body weight or any serum lipid measure. Collectively, these findings indicate that consumption of 7 commercially available eggs per week, which is in line with the current Australian dietary guidelines (17), represents a safe and effective dietary approach to attenuate the wintertime decrease in circulating 25(OH)D concentrations in young Australian adults residing in southern Australia.

The finding that consumption of 7 commercially available eggs per week, which provided a weekly vitamin D dose of ∼36 μg (∼1450 IU), was effective for reducing the wintertime decline in serum 25(OH)D concentrations compared with controls (2 eggs/wk) in our cohort of young adults, provides evidence that current natural feeding or fortification strategies used by egg producers in Australia can represent an effective approach to improve dietary vitamin D intakes. A previous 8-wk trial in Irish adults aged 45–70 y found that consumption of 7 vitamin D–enriched egg [cholecalciferol or 25(OH)D_3_ eggs] per week [providing 25–32 μg (990–1270 IU) vitamin D/wk] prevented the wintertime decrease in 25(OH)D concentrations (11). However, there are several differences in the findings from this trial and our study. First, in our study mean 25(OH)D concentrations decreased by 8.3 nmol/L (*P* = 0.061) or 9.7 nmol/L (*P* = 0.036) after excluding 1 participant who commenced taking vitamin D supplements, following the consumption of 7 eggs/wk for 12 wk. In contrast, in the earlier 8-wk Irish trial using eggs fortified with vitamin D–enriched feed, serum 25(OH)D concentrations remained unchanged [mean change: cholecalciferol eggs = 2.2 nmol/L; 25(OH)D_3_ eggs = −0.2 nmol/L]. Second, the mean wintertime decrease in serum 25(OH)D after 12 wk in the controls (2 eggs/wk) in our study was 28.6 nmol/L, which was considerably greater than the mean 6.4-nmol/L reduction after 8 wk reported in the controls (who habitually consumed ≤2 eggs/wk) in the Irish study (11). This is also greater than the mean 12–23-nmol/L wintertime reduction we have observed in previous Australian epidemiological studies (3, 5) and a 16-wk study involving Australian middle-aged women followed from summer/autumn to winter (24). These contrasting results could relate to differences in the age range (45–70 y compared with 25–40 y) and BMI (∼25 kg/m^2^ compared with 33) of participants, study duration (8 compared with 12 wk), and geographical location (latitude) and sun exposure habits, all of which are known to influence vitamin D status, and the higher mean baseline serum 25(OH)D concentrations in our study relative to the earlier Irish trial (74–84 compared with 41–49 nmol/L). For instance, previous research has shown that the serum 25(OH)D responses to vitamin D supplementation or vitamin D–fortified foods are influenced by baseline concentrations, with greater changes observed in those with lower initial 25(OH)D concentrations (15, 16, 25). However, this still does not explain why we observed a greater decrease in serum 25(OH)D concentrations in our study given that both trials used the same dose of eggs (*n* = 7/wk) that provided a similar weekly amount of vitamin D, although it is worth noting that our study was conducted during a lockdown period in Melbourne due to COVID-19.

Other factors known to contribute to differences (changes) in serum 25(OH)D concentrations include habitual dietary vitamin D intake and sun exposure habits. In our study, habitual vitamin D intakes could not be determined because there is no comprehensive food database available (or included in the ASA24 dietary platform) on the vitamin D content of foods in Australia. However, previous research has reported the average dietary vitamin D intake of Australian adults is ∼2–4 μg/d (6, 26). Based on this estimate, the total daily vitamin D intake of participants in the 7-eggs/wk group in our study would be 7–9 μg/d, which is comparable to the 9.5–10.4 μg/d (total vitamin intake) reported in the previous 8-wk intervention by Hayes and colleagues (11). In this study (as in our trial), all participants were instructed not to take vitamin D–containing supplements prior (4 to 12 wk) to the trial and throughout the study. Thus, it is unlikely that dietary vitamin D intakes contributed to the different findings. Because sun exposure is the main source of vitamin D, unique to our study is our assessment of habitual sun exposure and sun protection practices every 3 wk throughout the intervention. In Australia, current guidelines recommend 2–3 h/wk of sun exposure (face, arms, hands, or equivalent; 3–6 times this amount for dark skin) around midday from May to August in the southern states to meet vitamin D requirements(27). In our study, 71–82% of participants spent ≤30 min/d outdoors on the weekday between 10:00 and 14:00, which is the peak UV period during May to August, throughout the entire 12-wk study period. Although time spent outdoors during this time of the day increased on the weekends, the vast majority (69–82%) of participants spent ≤60 min/d outdoors on the weekend. In addition, nearly all (86–100%) participants indicated that they undertook safe sun protection practices at all times throughout the study. Thus, it is possible that this somewhat limited sun exposure and high use of safe sun protection practices contributed to the large decrease in serum 25(OH)D concentrations observed in the controls in our study.

To our knowledge, this is the first RCT to investigate the dose–response effect of eggs on serum 25(OH)D concentrations in younger adults. However, we observed no additional benefit of consuming 12 compared with 7 eggs/wk on serum 25(OH)D concentrations, despite the weekly dose of vitamin D increasing to ∼62 μg (∼2480 IU) for the 12-eggs/wk group, assuming a conversion factor of 5 for 25(OH)D_3_. It is difficult to explain these findings because adherence was similar for both groups (mean 83–86%) and previous research has shown that serum 25(OH)D concentrations increase by around 1–2 nmol/L for every additional 100 IU of vitamin D-3 (14), including with the use of vitamin D–fortified foods (9, 15, 16). However, as stated above our estimation of the total vitamin D content of the eggs included a conversion factor of 5 for the 25(OH)D_3_ content of the eggs (11). Others have reported lower conversion factors (∼1 to 3) (28, 29), which if applied to our 7- and 12-eggs/wk groups would translate to an additional 16.4–28.1 μg (656–1124 IU) vitamin D per week based on a conversion factor of 1, or 26.3–45.1 μg (1052–1804 IU) vitamin D per week based on a conversion factor of 3. Intakes of vitamin D at these levels (equivalent to 2.3–4.0 μg/d and 3.8–6.4 μg/d) are mostly below the current AI of 5 μg/d vitamin D for Australians aged 1–50 y (7). It has been acknowledged that the current guidelines for the AI of vitamin D in Australia are outdated (6), with intakes of 10–15 μg/d (400–600 IU/d) typically recommended to maintain adequate vitamin D status throughout the year (6, 8, 30). Despite the lack of an increase in serum 25(OH)D concentrations in either the 7- or 12-eggs/wk group in our study, the finding that there was a significant net benefit of ∼20 nmol/L relative to controls (2 eggs/wk) is in line with the findings from a previous meta-analysis reporting that vitamin D intakes of ∼11 μg/d (∼440 IU/d) from fortified foods resulted in a treatment effect of 19.4 nmol/L (16).

Given that consumption of 12 eggs/wk exceeds the recommendation of the Australian Dietary Guidelines of 7 eggs/wk (17), it was important to understand the participants’ acceptability to eating the different doses of eggs prescribed in this study. All participants rated highly that they liked eating the eggs and their taste, and that it was not too difficult or too much effort to consume their prescribed dose of eggs. Although there were no significant differences in acceptability scores between the 3 groups, there was a trend (*P* = 0.06) for participants in the 12-eggs/wk group to report lower scores compared with controls and the 7-eggs/wk group to the questions *“How well did you like the taste of eggs?* and *“How easy or difficult was it for you to prepare the eggs to eat?”* Acceptability scores for the controls and 7-eggs/wk group were almost identical, indicating that any future recommendations to consume 7 egg/wk to prevent the wintertime decline in 25(OH)D is likely to be regarded as acceptable by young adults. Finally, there were no group differences for the changes in mean body weight or any of the serum lipid concentrations, which is important to alleviate any potential concerns that 7 to 12 eggs/wk might have adverse health effects. However, it is important to acknowledge that our study was not powered to detect any potential differences for change in serum lipid concentrations, and thus these findings must be interpreted with caution and might not be generalizable to other cohorts. For instance, further research is warranted to evaluate the safety-risk profile of different doses of eggs in individuals with or at increased risk of cardiovascular disease (CVD) given that eggs are rich in choline, which can increase the microbially derived cardiotoxin trimethylamine-*N*-oxide (TMAO), a known risk factor for CVD (31). Nevertheless, our results are consistent with the findings from several previous trials over 8 to 12 wk showing that consumption of 7 or 12 eggs/wk did not have any adverse effects on the serum lipid profile when comparing changes between the groups (11, 32), although there was an increase in total cholesterol and LDL cholesterol in the 12-eggs/wk group.

The strengths of this study include: *1*) it is, to our knowledge, the first 3-arm RCT to evaluate the dose–response effects of consuming commercially available eggs on serum 25(OH)D concentrations; *2*) the comprehensive compliance monitoring and high adherence to the different doses of eggs; *3*) the 12-wk intervention period because circulating 25(OH)D concentrations tend to plateau around this time after vitamin D treatment (6); and *4*) the sampling of different batches of the same commercially available eggs from various locations at 2 timepoints to evaluate their vitamin D content. Limitations include: the lack of assessment of dietary vitamin D intake; the use of a self-report questionnaire to assess habitual sun exposure habits rather than a more accurate measure such as a UV exposure dosimeter; self-report of body weight, which was necessary due to COVID restrictions; and the failure to consider other relevant cardiovascular risk biomarkers, particularly TMAO, which is an independent risk factor for CVD (31). The high baseline serum 25(OH)D concentrations of participants is also a potential limitation given that the response to vitamin D treatment is typically greater in those with lower initial 25(OH)D concentrations (15, 16, 25).

In conclusion, this study indicates that consumption of 7 commercially available eggs per week, which is in line with the current Australian dietary guidelines (17), was safe, acceptable, and effective for attenuating the wintertime decrease in serum 25(OH)D concentrations in young Australian adults. Consuming 12 eggs/wk did not result in any added benefits to serum 25(OH)D concentrations over 7 eggs/wk. These findings indicate that weekly consumption of 7 eggs should be included as an important dietary approach to help to optimize vitamin D status during the winter months in Australia.

## Supplementary Material

nxac044_Supplemental_File

## Data Availability

Data described in the manuscript, code book, and analytic code will be made available from the corresponding author upon reasonable request.

## Notes

This study was supported by a grant from Australian Eggs. The sponsor was not involved in the conduct of the study, collection, management or analysis of the data, or the decision to publish the results.

Author disclosures: RMD and S-YT report a grant from Australian Eggs to conduct this study. All other authors report no conflicts of interest.

Supplemental Tables 1–3 are available from the “Supplementary data” link in the online posting of the article and from the same link in the online table of contents at https://academic.oup.com/jn/.

Abbreviations used: AI, adequate intake; ASA24, Automated Self-Administered 24-Hour Dietary Assessment Tool; CVD, cardiovascular disease; MVPA, moderate-vigorous physical activity; NATA, National Association of Testing Authorities; RCT, randomized controlled trial; TMAO, trimethylamine-*N*-oxide; 25(OH)D, 25-hydroxyvitamin D.
