# Supplementary material for: Dose–Response Effect of Consuming Commercially Available Eggs on Wintertime Serum 25-Hydroxyvitamin D Concentrations in Young Australian Adults: a 12-Week Randomized Controlled Trial
Source: J Nutr. 2022 Feb 26;152(7):1702–10. doi: 10.1093/jn/nxac044 (PMC9258551; doi:10.1093/jn/nxac044)
Supplement: nxac044_Supplemental_File [file nxac044_supplemental_file.docx]

Dose-Response Effect of Consuming Commercially Available Eggs on Wintertime Serum 25-hydroxyvitamin D Concentrations in Young Australian Adults: A 12 week Randomized Controlled Trial.

Daly RM Online Supplementary Material.

**Supplementary Table 1**: Vitamin D content of the commercial eggs used in the study.^1^

|  | **Vitamin D Content of Eggs (μg per 60g egg)** | | |
| --- | --- | --- | --- |
|  | **Cholecalciferol (vitamin D_3_)** | **25(OH)D_3_** | **Total Vitamin D Activity^2^** |
| Baseline | 1.82 ± 0.61 | 0.72 ± 0.16 | 5.42 ± 0.64 |
| Week 6 | 1.44 ± 0.87 | 0.70 ± 0.37 | 4.94 ± 2.42 |
| *Mean* | *1.63 ± 0.70* | *0.71 ± 0.25* | *5.18 ± 1.60* |

^1^ Values are means ± standard deviations, nine free-range eggs from three batches (60g/egg) purchased from three different supermarkets were combined for analysis at baseline and again at week 6. ^2^ Total vitamin activity was derived from the following formula: cholecalciferol (vitamin D_3_) + [5 x 25(OH)D_3_].

**Supplementary Table 2**: Mean dietary intakes and moderate-vigorous physical activity levels in the young adults at baseline and week 12 in the 2 (control), 7 and 12 eggs per week groups.^1^

|  | **n** | **Control** | **n** | **7 Eggs/week** | **n** | **12 Eggs/week** | *P-value* **^3^** |
| --- | --- | --- | --- | --- | --- | --- | --- |
| Energy, kJ/d |  |  |  |  |  |  |  |
| Baseline | 16 | 7574 ± 1902 | 16 | 8194 ± 1787 | 17 | 9031 ± 2700 | *0.638* |
| Week 12 | 11 | 7236 ± 1958 | 13 | 8364 ± 2033 | 13 | 8918 ± 2073 |  |
| *P-value ^2^* |  | *P=0.353* |  | *P=0.896* |  | *P=0.232* |  |
| Protein, g/d |  |  |  |  |  |  |  |
| Baseline | 16 | 76.9 ± 20.1 | 16 | 85.9 ± 29.7 | 17 | 81.0 ± 24.2 | *0.213* |
| Week 12 | 11 | 75.7 ± 33.2 | 13 | 103.9 ± 31.3 | 13 | 100.1 ± 24.7 |  |
| *P-value ^2^* |  | *P=0.846* |  | *P=0.187* |  | *P=0.029* |  |
| Carbohydrates, g/d |  |  |  |  |  |  |  |
| Baseline | 16 | 194 ± 57.5 | 16 | 196 ± 40.3 | 17 | 246 ± 98.4 | *0.844* |
| Week 12 | 11 | 175 ± 48.6 | 13 | 195 ± 40.3 | 13 | 211 ± 66.8 |  |
| *P-value ^2^* |  | *P=0.250* |  | *P=0.736* |  | *P=0.055* |  |
| Fat, g/d |  |  |  |  |  |  |  |
| Baseline | 16 | 72.8 ± 24.7 | 16 | 78.3 ± 23.6 | 17 | 84.7 ± 31.9 | *0.773* |
| Week 12 | 11 | 73.6 ± 30.3 | 13 | 77.1 ± 30.8 | 13 | 87.7 ± 26.1 |  |
| *P-value ^2^* |  | *P=0.692* |  | *P=0.728* |  | *P=0.803* |  |
| Saturated fat, g/d |  |  |  |  |  |  |  |
| Baseline | 16 | 25.4 ± 10.2 | 16 | 27.7 ± 9.7 | 17 | 28.2 ± 12.3 | *0.335* |
| Week 12 | 11 | 29.1 ± 14.6 | 13 | 24.5 ± 10.3 | 13 | 31.5 ± 13.8 |  |
| *P-value ^2^* |  | *P=0.291* |  | *P=0.295* |  | *P=0.969* |  |
| Physical activity, min/week |  |  |  |  |  |  |  |
| Baseline | 17 | 325 ± 178 | 17 | 379 ± 300 | 17 | 430 ± 374 | *0.392* |
| Week 12 | 13 | 238 ± 230 | 12 | 393 ± 321 | 15 | 347 ± 305 |  |
| *P-value ^2^* |  | *P=0.382* |  | *P=0.500* |  | *P=0.531* |  |

^1^ All values are unadjusted means ± standard deviations (SD)

^2^ Paired t-tests used to test for differences between baseline and week 12 values.

^3^ P-values for between group differences were assessed using ANCOVA adjusted for baseline values, age and sex.

**Supplementary Table 3**: Percentage of young adults in the 2 (control), 7 and 12 eggs per week group that spent different times outdoors on the weekdays and weekend days between 10am and 2pm at baseline, 3, 6, 9 and 12 weeks.^1^

|  | **Weekdays (10am-2pm)** | | | | **Weekend days (10am-2pm)** | | | |
| --- | --- | --- | --- | --- | --- | --- | --- | --- |
|  | *<15* | *15-30* | *30-60* | *>60* | *<15* | *15-30* | *30-60* | *>60* |
| **Baseline** |  |  |  |  |  |  |  |  |
| Controls | 30% | 35% | 35% | 0% | 12% | 29% | 47% | 12% |
| 7 Eggs/week | 30% | 35% | 35% | 0 | 12% | 12% | 35% | 41% |
| 12 Eggs/week | 47% | 35% | 6% | 12% | 6% | 35% | 18% | 41% |
| *Chi-square* | *P=0.22* | | | | *P=0.09* | | | |
| **Week 3** |  |  |  |  |  |  |  |  |
| Controls | 40% | 27% | 33% | 0% | 13% | 27% | 47% | 13% |
| 7 Eggs/week | 25% | 38% | 25% | 12% | 0% | 38% | 31% | 31% |
| 12 Eggs/week | 50% | 38% | 6% | 6% | 6% | 35% | 18% | 41% |
| *Chi-square* | *P=0.38* | | | | *P=0.61* | | | |
| **Week 6** |  |  |  |  |  |  |  |  |
| Controls | 40% | 33% | 20% | 7% | 33% | 27% | 47% | 13% |
| 7 Eggs/week | 40% | 33% | 20% | 7% | 20% | 13% | 27% | 40% |
| 12 Eggs/week | 53% | 47% | 0% | 0% | 7% | 60% | 13% | 20% |
| *Chi-square* | *P=0.56* | | | | *P=0.05* | | | |
| **Week 9** |  |  |  |  |  |  |  |  |
| Controls | 47% | 20% | 26% | 7% | 13% | 27% | 33% | 27% |
| 7 Eggs/week | 27% | 33% | 33% | 7% | 7% | 13% | 53% | 27% |
| 12 Eggs/week | 50% | 43% | 7% | 0% | 7% | 50% | 21% | 22% |
| *Chi-square* | *P=0.47* | | | | *P=0.29* | | | |
| **Week 12** |  |  |  |  |  |  |  |  |
| Controls | 36% | 43% | 0% | 21% | 21% | 21% | 21% | 37% |
| 7 Eggs/week | 42% | 25% | 25% | 8% | 8% | 17% | 50% | 25% |
| 12 Eggs/week | 38% | 63% | 0% | 0% | 6% | 31% | 38% | 25% |
| *Chi-square* | *P=0.04* | | | | *P=0.62* | | | |

^1^ All values are percentage.
